# New insights into the mechanism of dynein motor regulation by lissencephaly-1

**DOI:** 10.7554/eLife.59737

**Published:** 2020-07-21

**Authors:** Steven M Markus, Matthew G Marzo, Richard J McKenney

**Affiliations:** 1 Department of Biochemistry and Molecular Biology, Colorado State UniversityFort CollinsUnited States; 2 Department of Molecular and Cellular Biology, University of California, DavisDavisUnited States; MRC Laboratory of Molecular BiologyUnited Kingdom; Utrecht UniversityNetherlands

**Keywords:** dynein, LIS1, lissencephaly, dynactin, nuclear migration, neuronal migration

## Abstract

Lissencephaly (‘smooth brain’) is a severe brain disease associated with numerous symptoms, including cognitive impairment, and shortened lifespan. The main causative gene of this disease – lissencephaly-1 (LIS1) – has been a focus of intense scrutiny since its first identification almost 30 years ago. LIS1 is a critical regulator of the microtubule motor cytoplasmic dynein, which transports numerous cargoes throughout the cell, and is a key effector of nuclear and neuronal transport during brain development. Here, we review the role of LIS1 in cellular dynein function and discuss recent key findings that have revealed a new mechanism by which this molecule influences dynein-mediated transport. In addition to reconciling prior observations with this new model for LIS1 function, we also discuss phylogenetic data that suggest that LIS1 may have coevolved with an autoinhibitory mode of cytoplasmic dynein regulation.

## LIS1 is a critical effector of human brain development

Lissencephaly is a severe developmental human brain disease that is characterized by agyria – a lack of convolutions known as gyri or sulci – and a brain with a resulting smooth cerebral surface. This disease, which affects approximately 1 in 30,000 individuals (Dobyns et al., 1993; Reiner et al., 1993), is associated with severe cognitive and motor function impairment, epilepsy, and a shortened lifespan (Guerrini and Parrini, 2010). Classical lissencephaly is characterized by disorganization of the neuronal tissue architecture found in healthy individuals: instead of the well-defined six neuronal layers formed in normal brains, those from lissencephalic patients typically exhibit four disordered layers (Golden, 2018).

**Figure 1.**
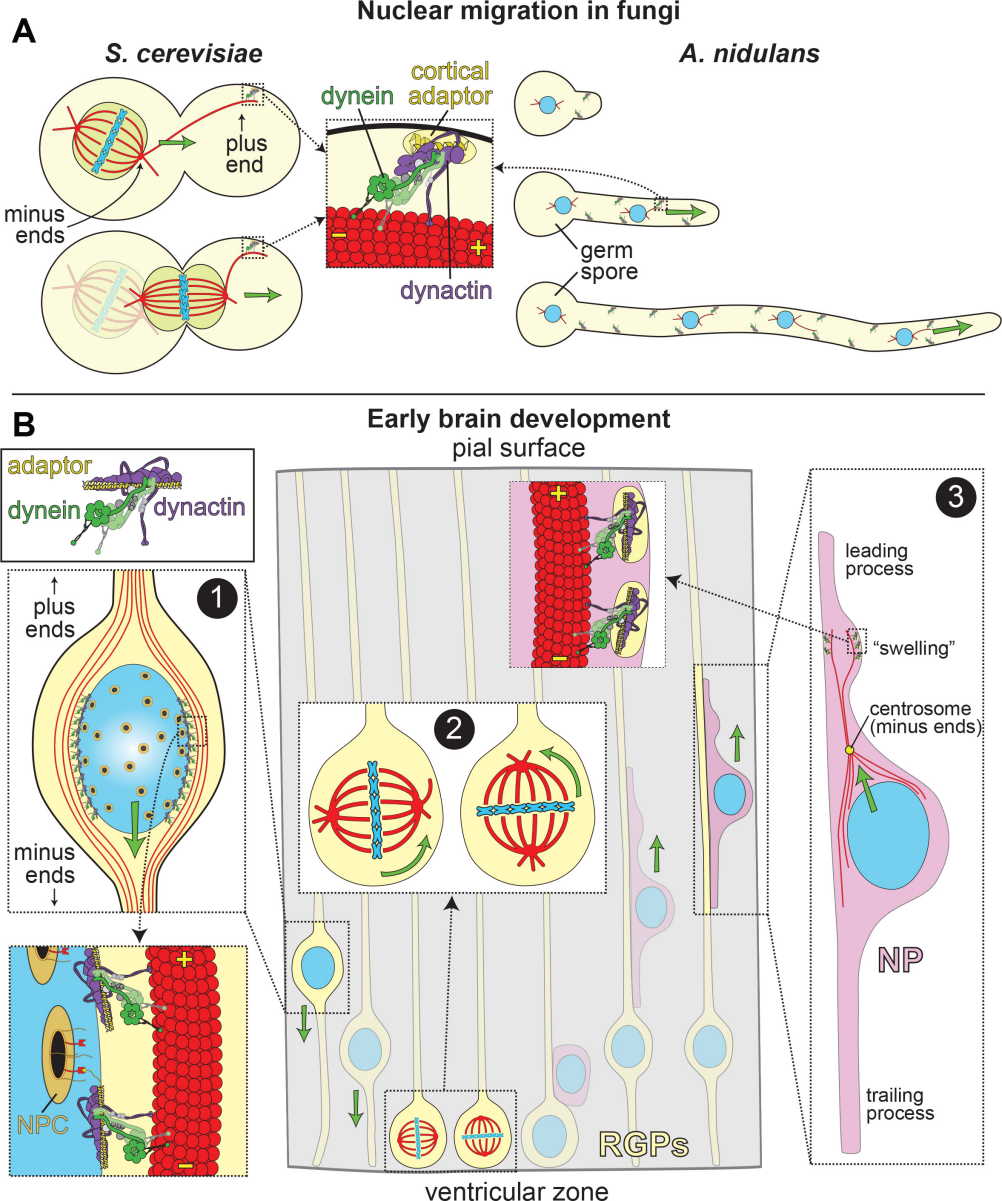
LIS1 and Dynein ensure appropriate transport and position of nuclei in various biological systems. (**A**) In fungal model systems, cortically anchored dynein-dynactin complexes move the nucleus toward the mother-bud neck (the future site of cytokinesis) in budding yeast (left), or in the direction of the growing hyphal tip to ensure proper nuclear distribution in *Aspergillus nidulans* (right). Dynein-dynactin complexes are recruited and anchored to the cell cortex in budding yeast and *A. nidulans* by Num1 and ApsA, respectively, (Fischer and Timberlake, 1995; Heil-Chapdelaine et al., 2000; Veith et al., 2005), which exhibit significant degrees of similarity within their C-terminal pleckstrin homology domains (for membrane association; 50% identity/64% similarity) and their N-terminal coiled-coil-containing dynein-dynactin interacting regions (24% identity/41% similarity; note this has not been experimentally determined in *A. nidulans*) (Tang et al., 2012). (**B**) In the developing brain, LIS1 and dynein are critical for the three distinct illustrated processes: (Box 1) During interkinetic nuclear migration (INM), the nucleus is transported to the ventricular surface in highly elongated radial glial precursors (RGPs), where mitosis takes place. Dynein-dynactin complexes anchored to the nuclear envelope – through interactions between dynein adaptors (*e.g.*, BicD2, in yellow) and nuclear pore complex components (*e.g.*, RanBP2, in red; nuclear pore complex, or NPC, in orange) – transport the nucleus toward the minus ends of microtubules (in red), which are situated toward the ventricular surface (‘-' and ‘+' depict minus and plus ends of microtubules, respectively). (Box 2) The mitotic spindles (in red, with chromosomes shown in blue) in RGPs are actively rotated by cortical dynein-dynactin complexes. The orientation of the spindle dictates whether a dividing cell will generate two RGPs (horizontal orientation, left), or one RGP and a differentiated cell type (*e.g.*, a neuronal precursor, or NP cell; vertical, right). (Box 3) Subsequent to their generation from RGPs, NPs ascend toward the pial surface of the developing brain, where the cortical plate resides. Evidence suggests that dynein is enriched in a dilation, or ‘swelling’ within the leading process. It has been proposed that dynein anchored at the cell cortex (by an unknown cortical receptor, in yellow) in this region pulls on astral microtubules emanating from the centrosome, thus moving the nucleus toward the leading process (see text) (Tsai et al., 2007). The manner by which the centrosome is linked to the nucleus is not entirely clear, but may rely on dynein and microtubules (Vallee et al., 2009).

Like other developmental brain disorders (*e.g.*, subcortical band heterotopia, focal cortical dysplasia) that result in reduced gyration, cell mass, or well organized tissue layers, lissencephaly is thought to be largely a result of defective neuronal migration (Guerrini and Parrini, 2010). The precursor cells for the majority of neurons and glia in the developed neocortex are radial glial progenitors (RGPs), highly elongated neuroepithelial cells that span the neural tube and developing cortex, from the ventricular to the pial surface (Figure 1B). The cell bodies for these key neuronal progenitors are found in the ventricular zone (VZ). Following an asymmetric cell division that results in a new RGP cell and a differentiated neuronal cell type (*e.g.*, a neuronal precursor, or NP), the latter cells undergo extensive migration toward the pial surface, ultimately occupying positions within the cortical plate, the precursor to the cerebral cortex (Vallee et al., 2009). Thus, mutations in the molecular effectors of this process result in lissencephaly or related disorders.

Unsurprisingly, this complex developmental process involves many molecular players. These include factors that affect microtubule function either indirectly (RELN, a serine protease [Hong et al., 2000]), or directly (TUB1A1, which encodes α-tubulin [Poirier et al., 2007]). However, the primary genetic drivers of lissencephaly are missense mutations in, or truncations and deletions of the LIS1 gene (lissencephaly-1) (Reiner et al., 1993), which was confirmed to be required for neuronal migration in a mouse model (Hirotsune et al., 1998), and in fly embryos (Swan et al., 1999). As a consequence of its central roles in lissencephaly and brain development, LIS1 has been a focus of intense scrutiny ever since its discovery 27 years ago. These studies have revealed invaluable insight into the cellular and biochemical roles for this molecule in neuronal function. LIS1 was initially identified as a subunit of brain platelet-activating factor acetylhydrolase (beta subunit, or PAF-AH1B1) (Hattori et al., 1994) and can indeed modulate PAF-AH enzyme activity in vitro; however, superstoichiometric amounts of LIS1 are needed to do so (Manya et al., 1999). Mice lacking PAF-AH alpha subunits do not exhibit defects in brain development (Koizumi et al., 2003; Yan et al., 2003), suggesting that this function of LIS1 is not responsible for the lissencephaly phenotype. Subsequently, numerous studies have since shown that LIS1 is also a key effector of the molecular motor cytoplasmic dynein. Given the well-defined role for the dynein complex in effecting microtubule-based transport – and in neuronal migration – the molecular basis for LIS1-related lissencephalies is likely defective dynein-based transport.

In this review, we summarize the cell biological and genetic data defining the role for LIS1 in cell and organismal function, and the wealth of biochemical data that have shed invaluable light on the mechanism by which this molecule functions. In addition to describing a phylogenetic relationship between LIS1 and dynein autoinhibition, we will attempt to reconcile the numerous – and at times contradictory – biochemical findings with in vivo data to synthesize them into a coherent model for LIS1 function in the dynein pathway.

## Dynein motor activity is complex and highly regulated

Cytoplasmic dynein-1 (hereafter referred to as dynein) is a minus end-directed microtubule motor complex comprised of several peptides, with the heavy chain – encoded by DYNC1H1 in humans – being responsible for the catalytic and microtubule-binding activities (reviewed in Schmidt and Carter, 2016). Dynein effects the majority of retrograde transport in organisms throughout the evolutionary spectrum, except in the plant kingdom, which has lost the cytoplasmic dynein machinery (Wickstead and Gull, 2007). Unlike the kinesin superfamily, which is composed of ~45 members in humans, there are only two genes encoding the cytoplasmic dynein heavy chains: DYNC1H1, and DYNC1H2, the latter of which encodes the heavy chain for cytoplasmic dynein-2, a motor restricted to ciliary and flagellar transport (*i.e.*, intraflagellar transport, or IFT). Also unlike some kinesin family members, metazoan dynein is unable to move processively along microtubules without the assistance of other factors (McKenney et al., 2014; Schlager et al., 2014). These include the multi-subunit complex dynactin, and any one of a number of identified cargo adaptor proteins that mediate an interaction between dynein and dynactin (*e.g.*, BicD2, BicDR1, Hook3, *etc.*) (reviewed in Reck-Peterson et al., 2018).

Recent elegant in vitro work has revealed the likely structural and biochemical basis for dynein’s reliance on these factors. Isolated dynein adopts an autoinhibited conformational state (called the ‘phi’ particle, due to its similar appearance to the Greek letter [Amos, 1989]) that reduces its microtubule on-rate, and also restricts its ability to interact with dynactin and an adaptor (Zhang et al., 2017). Upon adopting the ‘open’ (uninhibited) state, dynein readily binds to both, and assembles into a motility competent dynein-dynactin-adaptor (DDA) complex (Zhang et al., 2017). Dynactin helps activate dynein motility by promoting microtubule binding of the DDA complex, and by orienting the motor heads of dynein in a parallel manner that is conducive to motility (McKenney et al., 2016; Zhang et al., 2017).

The reliance of dynein on these factors for processive motility provides the ability to fine-tune dynein-mediated transport within the cell. For example, different cargo adaptors can provide cargo specificity by recruiting dynein-dynactin complexes to different structures or regions of the cell. Moreover, those factors that promote DDA assembly enhance dynein activity, while those that prevent it have the opposite effect. Since the discovery of the autoinhibited state and its role in affecting dynein activity is fairly new, it is not surprising that the field is currently focused on characterizing factors that may affect it. Recent studies have revealed that LIS1 is in fact a key effector that promotes and/or stabilizes the uninhibited ‘open’ conformation, thus promoting DDA assembly and motility (discussed below).

## Dynein and LIS1 are key effectors of spindle positioning and nuclear migration

The mechanism by which neurons undergo extensive migration from the ventricular zone to the pial surface has been examined in detail (Figure 1B). Similar to other forms of cell migration, neuronal migration appears to follow a three-step process: (1) the cell extends a leading process, which is a dynein-independent event (Tsai et al., 2007; Tsai et al., 2005); (2) the nucleus migrates into the leading process (‘nucleokinesis’); (3) the trailing process is retracted (Lambert de Rouvroit and Goffinet, 2001). Thus, nucleokinesis, or nuclear migration, is a key process in cell migration. Determining the mechanisms by which dynein effects this process – in which it plays a central role – is important to understand how LIS1 dysfunction leads to lissencephaly.

A role for dynein in effecting nuclear movements was first reported in budding yeast and filamentous fungi. Budding yeast mutants lacking a dynein heavy chain exhibited a binuclear phenotype (Eshel et al., 1993; Li et al., 1993), while *Aspergillus nidulans* and *Neurospora crassa* dynein mutants exhibited a nuclear distribution phenotype (Plamann et al., 1994; Xiang et al., 1994) (leading to the *NUD* names applied to genes implicated in this process [Morris, 1975]), in which the nuclei are aberrantly clustered at one end of the germ spore instead of being evenly distributed along the hyphae. In budding yeast, the nucleus, which is initially positioned within the mother cell, must be actively positioned to the site of cytokinesis (the mother-bud neck), such that at the moment of anaphase onset, the chromosomes are equally segregated to mother and daughter cells (Figure 1A, left) (Markus et al., 2012; Moore et al., 2009). In *A. nidulans*, an asexual spore containing a single nucleus undergoes polarized growth to form a germling (or germ tube). During this process, nuclear division occurs simultaneously with nuclear translocation into the germ tube (Figure 1A, right). In both model systems, nuclear migration relies on dynein pathway components and many of its regulators, including dynactin and LIS1 (Pac1 in *S. cerevisiae*, which possesses 27% identity and 44% similarity with LIS1; NudF in *A. nidulans*, which possesses 43% identity and 62% similarity with LIS1) (Eshel et al., 1993; Lee et al., 2003; Li et al., 1993; Moore et al., 2008; Xiang et al., 1994; Xiang et al., 1995; Xiang et al., 1999; Zhang et al., 2008).

Dynein-mediated nuclear migration is perhaps best understood in budding yeast, owing in part to the simplicity of the system, a well-defined ‘parts list’, and the fact that the only known transport function for dynein in this organism is to translocate the nucleus with the enclosed mitotic spindle (as a result of the ‘closed mitosis’ that takes place in this organism; Figure 1A, left). Numerous studies from several labs have provided evidence for the prevailing ‘offloading’ model for dynein function in this organism. In this model, dynein binds to Pac1/LIS1 in the cytoplasm, and the resulting dynein-Pac1 complex subsequently binds to Bik1 (homolog of human CLIP-170) that is associated with dynamic microtubule plus ends (by an as yet unknown EB1-independent mechanism) (Carvalho et al., 2004; Lee et al., 2005; Lee et al., 2003; Markus and Lee, 2011; Markus et al., 2011; Sheeman et al., 2003). Data indicate that this plus end-bound dynein pool recruits the dynactin complex, which is then competent for interaction with the cortical receptor, Num1 (Heil-Chapdelaine et al., 2000; Markus et al., 2011; Moore et al., 2008). Upon encountering cortically-anchored Num1, the plus end-bound dynein-dynactin complex is transferred, or ‘offloaded,’ and activated for effecting nuclear migration. Although the role of Num1 in this process is not entirely clear, it likely plays a role analogous to mammalian cargo adaptor proteins in that it promotes a stable, motility-competent dynein-dynactin complex (Lammers and Markus, 2015). It is interesting to note that in this system, dynactin appears to associate with the plus end-bound dynein complex before encountering the cargo adapter molecule Num1. Similar plus end localization of dynein and dynactin has been reported in mammalian cells and filamentous fungi (Han et al., 2001; Splinter et al., 2012; Valetti et al., 1999; Vaughan et al., 1999; Zhang et al., 2003), which has been proposed to precede cargo adaptor binding, and consequent activation of minus end motility (Baumbach et al., 2017; Egan et al., 2012; Jha et al., 2017; Lenz et al., 2006; Moon et al., 2014; Schmidt et al., 2017; Zhang et al., 2010). Thus, dynein and dynactin appear to have the capacity to interact in a cargo adaptor-independent manner – at least in the context of plus end-targeting – a phenomenon that is distinct from what is observed in single-molecule motility assays (McKenney et al., 2014; Schlager et al., 2014). Further work is needed to explore the nature of this unique adaptor-independent dynein-dynactin complex, but we speculate that the well-defined interaction between the dynein intermediate chain (DIC) and the p150 subunit of dynactin are likely important (Karki and Holzbaur, 1995; McKenney et al., 2011; Nyarko et al., 2012; Stuchell-Brereton et al., 2011; Vaughan and Vallee, 1995).

Before a role for dynein in nuclear migration was apparent in higher eukaryotes, it was demonstrated that dynein could effect the position of the mitotic spindle in cultured epithelial (MDCK) cells (Busson et al., 1998). Numerous studies have also shown that cortically anchored dynein-dynactin plays a critical role in orienting the mitotic spindle in a manner analogous to budding yeast (Kiyomitsu and Cheeseman, 2012; Kiyomitsu and Cheeseman, 2013; O'Connell and Wang, 2000; Okumura et al., 2018; Peyre et al., 2011; Woodard et al., 2010). The position of the mitotic spindle in animal cells is indeed nonrandom, and is precisely tuned to match the specific needs of a cell and its surrounding tissue. The position of the spindle dictates the position of the cytokinetic contractile ring machinery; thus, by positioning the spindle offset from the cell center, the cell divides asymmetrically. In fact, directed positioning (*e.g.*, center, or offset from center) or orientation of the spindle (*e.g.*, parallel with, or orthogonal to the plane of the tissue) can dictate the fate of a cell (reviewed in [Knoblich, 2010; Morin and Bellaïche, 2011]) in part by asymmetrically distributing cellular factors to the resulting daughter cells. Given a reported role for LIS1 in dynein-mediated spindle orientation (Faulkner et al., 2000; Pawlisz et al., 2008; Siller and Doe, 2009; Xie et al., 2013; Yingling et al., 2008), and the importance of spindle orientation in neuronal cell differentiation and generation of progenitors (Bershteyn et al., 2017; Fish et al., 2006; Götz and Huttner, 2005), another potential confounding cause for lissencephaly is dysfunctional spindle orientation, in addition to defects in nuclear migration (Figure 1B; see Box 2).

A potential role for dynein in nuclear migration in metazoans was suggested from wound healing studies in cultured fibroblasts. During the healing process, cells at the wound edge reorient their centrosome (the microtubule organizing center) toward the wound prior to cell migration in this direction. Dynein and dynactin are both required for reorientation of the centrosome (Etienne-Manneville and Hall, 2001; Palazzo et al., 2001) – which precedes nuclear movements – and are also required for directed cell migration (Dujardin et al., 2003). Of note, dynein and LIS1 both accumulate at the leading edge of these migrating fibroblasts, implicating these cortically anchored molecules in directing centrosomal movement. In a similar process, the centrosome also precedes the nucleus during nuclear migration in neurons (Figure 1B, Box 3; Solecki et al., 2004), albeit in a manner that indicates they are not directly coupled. Notably, depletion of either dynein or LIS1 in embryonic neural precursor cells is sufficient to block nuclear movement (Tsai et al., 2007). Although the mechanism by which dynein and LIS1 effect nuclear movement in these radially migrating neocortical neurons is not entirely clear, the centrosome typically moves toward, and pauses when it reaches a ‘swelling’ – a bulge in the leading process that often appears, prior to nuclear movement (Figure 1B; Box 3). Fluorescence microscopy revealed an enrichment of dynein in this swelling, as well as a polar network of microtubules emanating from the centrosome with their plus ends oriented toward the leading process (Tsai et al., 2007). This raised the possibility that dynein anchored at the plasma membrane of the swelling pulls the nucleus-attached centrosome toward it via its minus end-directed activity. The actin-based motor myosin has also been implicated in supporting these nuclear movements; however, while inhibition of myosin reduced nuclear movement, it did not prevent advancement of the centrosome (Tsai et al., 2007). Evidence indicates that myosin effects nuclear movement from behind the nucleus (Bellion et al., 2005; Schaar and McConnell, 2005), rather than in front, as is the case for dynein. Thus, dynein likely mediates centrosome advancement, while actomyosin-based processes are required for nuclear movements. It has also been speculated that dynein effects nuclear movements from the nuclear surface (Vallee et al., 2009); given the separation between the centrosome and the nucleus (by up to 15 µm), minus end-directed motility of nuclear envelope-anchored dynein motors would conceivably also result in migration toward the leading process.

Evidence for dynein motor activity localized to the nuclear envelope is more apparent in RGP cells, in which the nucleus undergoes an oscillatory migratory behavior termed interkinetic nuclear migration (INM). In a process that relies on dynein and LIS1, the nucleus is translocated down toward the ventricular surface where mitosis takes place (Figure 1B; see Box 1). Movement in the opposite direction – towards the basal surface, where the nucleus undergoes S phase – is mediated by the kinesin-3 Kif1A (Tsai et al., 2010). At least two independent mechanisms recruit dynein and dynactin to the nuclear envelope: the cargo adaptor protein BicD2, which is tethered by the nucleoporin RanBP2 (Splinter et al., 2012; Splinter et al., 2010); and, the kinetochore protein CENP-F, which interacts with dynein via the paralogous proteins Nde1/Ndel1, and is tethered by the nucleoporin Nup133 (Bolhy et al., 2011). These pools of dynein independently effect nuclear migration during INM (Baffet et al., 2015; Hu et al., 2013). Thus, dynein is a key effector of nuclear migration during early stages of brain development, which is critical for cell cycle progression of RGPs, and the consequent generation of neurons and glia that ultimately comprise the adult brain.

It is worth noting that in addition to a role in effecting nuclear migration, dynein and LIS1 have also been implicated in promoting nuclear envelope breakdown at the onset of mitosis (Hebbar et al., 2008; Salina et al., 2002; Turgay et al., 2014). In fact, heterozygous LIS1 null mice exhibit delayed nuclear envelope breakdown, and a consequent increased fraction of progenitor cells near the VZ undergoing mitosis (with respect to wild-type mice), providing yet another potential LIS1-based mechanism to disrupt neurogenesis (Hebbar et al., 2008). Although the mechanisms by which dynein and LIS1 effect this process are not entirely clear, evidence indicates that dynein-dynactin recruitment to the nuclear envelope is mediated by the LINC (linker of nucleoskeleton and cytoskeleton) complex, which is comprised of SUN (Sad1p and UNC-84 homology) and KASH (Klarsicht/Anc-1/SYNE homology) family proteins. The LINC complex has also been implicated in nuclear migration, most notably in *C. elegans* (reviewed in Bone and Starr, 2016; Tapley and Starr, 2013).

In summary, dynein is a critical effector of nuclear migration, which is intimately linked to neuronal migration (the former throughout the evolutionary spectrum). Both processes are critical during brain development and other important physiological events. Although the role for dynactin in this process is likely to promote a motility-competent dynein complex, the role of LIS1 is less clear. However, recent studies have shed new light on the mechanism by which LIS1 impacts dynein function, and provide a model by which LIS1 dysfunction might lead to disease.

## Conservation and structure of LIS1, and its interaction with dynein

**Figure 2.**
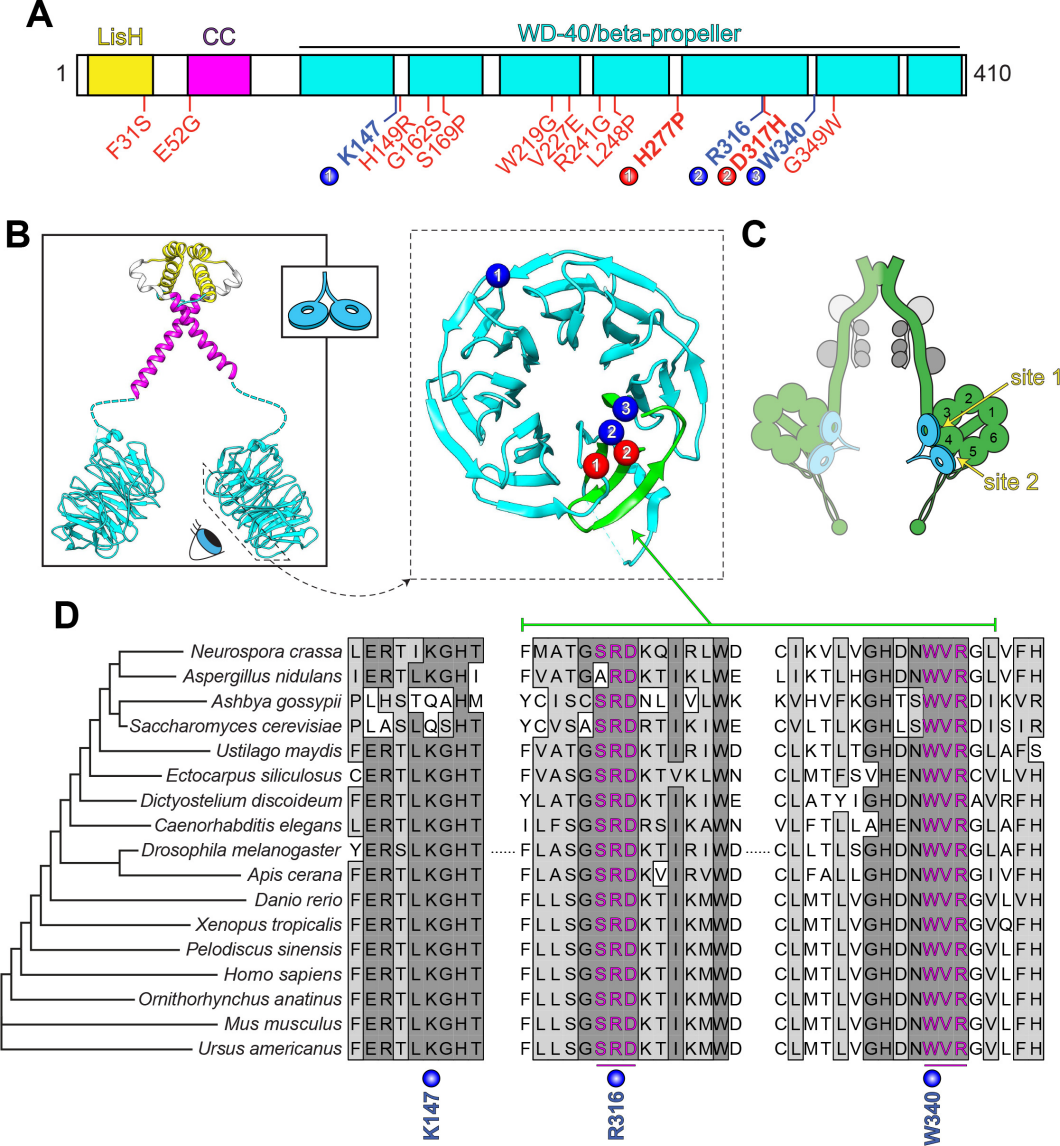
LIS1 structure and conservation. (**A**) Domain architecture of human LIS1. LIS1-homology (LisH), coiled-coil (CC), and WD-40 domains indicated, along with missense mutations found in patients with lissencephaly (red; bolded residues depicted in panel B, right) and the three residues shown to be important for Pac1/LIS1-dynein interaction (blue; numbers correspond to numbered residues in panel B, right) (Gutierrez et al., 2017; Pandey and Smith, 2011; Toropova et al., 2014). (**B**) Model of full-length, dimeric LIS1 structure (adapted from Tarricone et al., 2004), with domains color-coded according to panel A (generated using PDB 1VYH and 1UUJ [Kim et al., 2004; Tarricone et al., 2004] and UCSF Chimera [Pettersen et al., 2004]). Inset shows cartoon depiction of LIS1 used throughout this review. The dynein-interacting surface of one of the beta-propellers of LIS1 is shown, along with the residues found to be important for dynein binding (blue), and the surface-exposed lissencephaly-causing mutations (red). The 5^th^ blade of the beta-propeller that encompasses the highly conserved dynein-interacting residues is shown in green. (**C**) A cartoon schematic of the dynein complex (green, heavy chains with AAA+ domains numbered; gray, light, light-intermediate, and intermediate chains) with a LIS1 dimer bound to each motor head. Note that ‘site 1’ is a key site for the dynein-LIS1/Pac1 interaction (Gutierrez et al., 2017; Toropova et al., 2014), while ‘site 2’ has also been implicated in regulating dynein (DeSantis et al., 2017) (see text). It is also worth noting that multi-color single-molecule imaging experiments revealed that the majority of motile mammalian DDA or yeast dynein complexes, stably bind only one LIS1/Pac1 dimer (presumably bound to a single motor domain), although the binding of two LIS1 dimers to motile DDA or yeast dynein complexes has also been observed (Gutierrez et al., 2017; Marzo et al., 2020). (**D**) Sequence alignment of the dynein-interacting region of LIS1 (indicated in green in panel B, right) and its homologs from the indicated species. Note the presence of two small groups (three amino acids each) of nearly invariant residues (in magenta) that cluster around the two residues shown to be important for binding to dynein and PAF-AH (Gutierrez et al., 2017; Tarricone et al., 2004; Toropova et al., 2014).

LIS1 is a well-conserved protein that possesses two distinct functional domains: a short N-terminal region comprised of a short coiled-coil and a LIS1-homology, or LisH, domain (found in over 100 eukaryotic proteins [Emes and Ponting, 2001]), followed by a larger C-terminal domain comprised of seven WD-40 repeats, which are typical of seven-bladed beta-propeller structures (Figure 2A and B). WD-40 domains often function as multi-protein interaction platforms, suggesting this surface could act as a scaffold for recruitment of various LIS1-interacting regulators and targets (Schapira et al., 2017). The N-terminal region is important for LIS1 dimerization (Ahn and Morris, 2001; Kim et al., 2004), while the beta-propeller domain interacts directly with dynein, PAF-AH, and the dynein regulator Nde1/Ndel1 (Sasaki et al., 2000; Tai et al., 2002; Tarricone et al., 2004; Toropova et al., 2014). Although a crystal structure of the full-length molecule remains to be determined, individual structures of the N- and C-terminal regions reveal a general overview of the LIS1 dimer (Figure 2B, left) (Kim et al., 2004; Tarricone et al., 2004). Mutagenesis studies combined with binding assays revealed at least two regions of one face of the beta-propeller that make contacts with dynein (Figure 2B, right; mutations in blue residues each lead to disrupted dynein binding; note that blue residue 1 is not as well conserved as residues 2 and 3) (Gutierrez et al., 2017; Pandey and Smith, 2011; Toropova et al., 2014). Cryoelectron microscopy (cryoEM) studies have further identified one key site of interaction within the dynein AAA+ ring (near AAA3; Figure 2C; ‘site 1’) that is required for dynein-Pac1 binding, with a secondary site that has been proposed to be important for tuning dynein function (‘site 2’; discussed below) (DeSantis et al., 2017; Toropova et al., 2014). Sequence alignment reveals a high degree of conservation for the C-terminal dynein-binding region of LIS1 (Figure 2D; R316 and W340), with two small clusters of nearly invariant residues surrounding those shown to be important for this interaction (Figure 2D, magenta residues).

Although many of the lissencephaly-causing mutations result in truncated protein products (Cardoso et al., 2002; Fogli et al., 1999; Pilz et al., 1998; Uyanik et al., 2007), numerous cases of lissencephaly arise as a consequence of missense mutations that are scattered throughout the protein (Figure 2A, red residues) (Cardoso et al., 2002; Di Donato et al., 2018; Lo Nigro et al., 1997; Pilz et al., 1998; Torres et al., 2004). Of those mutations that are found within the beta-propeller region, only two map to the dynein-binding region (Figure 2A and B, red residues); the rest are buried within the beta-propeller structure, and likely lead to disease by destabilizing LIS1 structure (Caspi et al., 2003). However, both of the mutations within the dynein binding region were also shown to result in disruption of LIS1 structure and/or protein aggregation (Caspi et al., 2003; Gutierrez et al., 2017).

## Contrasting models for LIS1 function in dynein motility

The first indication that LIS1 may participate in the dynein pathway, independently of its role as a subunit of the PAF-AH complex, was the finding of similar nuclear migration phenotypes in LIS1 (NUDF) and dynein (NUDA) *A. nidulans* mutants (Xiang et al., 1995). Further arguing against a role for PAF-AH in neuronal development, deletion of the catalytic alpha subunit of PAF-AH leads to defects in testicular, not brain development (Koizumi et al., 2003; Yan et al., 2003). Additionally, there are no clear PAF-AH subunits in some organisms with LIS1 homologues (*e.g.*, fungi), arguing for an independent role of LIS1 in orthogonal pathways. Subsequent studies using cultured mammalian cells confirmed an interaction between LIS1 and dynein, and noted, among other things, an inward redistribution of dynein and dynactin to the centrosome (where microtubule minus ends are anchored) (Faulkner et al., 2000; Smith et al., 2000), and an incorporation of dynein and dynactin into higher molecular weight complexes upon LIS1 overexpression. Given the reliance of dynein on dynactin for processive motility, these data were the first indications that LIS1 may in fact promote assembly of dynein-dynactin complexes, thereby activating dynein motility (see below).

As described above, LIS1 is important for many dynein-mediated vesicular, nuclear, and neuronal migratory processes in lower and higher eukaryotes. However, the mechanism by which it functions has been controversial. What is clear is that LIS1 is important for recruitment of dynein-dynactin to various cellular structures, including: the nuclear envelope (Raaijmakers et al., 2013; Splinter et al., 2012), microtubule plus ends (Coquelle et al., 2002; Faulkner et al., 2000; Lee et al., 2003; Markus et al., 2009; Moon et al., 2014; Sheeman et al., 2003; Splinter et al., 2012), Rab6-positive vesicles (Splinter et al., 2012), kinetochores (Moon et al., 2014; Raaijmakers et al., 2013), the cell cortex (Markus et al., 2009; Moon et al., 2014), and ribonucleoprotein (RNP) complexes (Dix et al., 2013). As described above, LIS1-mediated targeting of dynein to the plus ends of dynamic microtubules has been implicated in delivering (or offloading) dynein-dynactin complexes to the cell cortex. This is best understood in budding yeast (Lee et al., 2005; Lee et al., 2003; Markus and Lee, 2011; Markus et al., 2011; Markus et al., 2009; Sheeman et al., 2003), although evidence suggests a similar mode of cortical delivery in *C. elegans* embryos and animal cells (Moon et al., 2014; Schmidt et al., 2017; Tame et al., 2014). A LIS1-dependent offloading mechanism has also been suggested for plus end-mediated delivery of dynein to vesicular cargo prior to their minus end-directed transport in filamentous fungi (Egan et al., 2012; Lenz et al., 2006). However, such an offloading mechanism for dynein recruitment is not universal. For instance, depolymerization of microtubules does not perturb dynein localization to kinetochores; rather, dynein accumulates to higher than normal levels due to the lack of kinetochore-microtubule attachments (Hoffman et al., 2001), which are ‘sensed’ by mitotic checkpoint machinery to ensure accurate chromosome segregation (reviewed in Foley and Kapoor, 2013; Sacristan and Kops, 2015).

Given the importance of LIS1 in dynein function, and the myriad cellular and developmental processes for which it is required, the precise molecular and biochemical basis by which LIS1 affects dynein function has been the focus of intense scrutiny in the field. These studies have yielded invaluable insight into LIS1 function; however, many of these findings have been contradictory, making it difficult to generate a unified hypothesis for LIS1 function. Most notably, evidence from several studies suggested that LIS1 inhibits dynein motor activity, while other work suggested the opposite: that LIS1 activates dynein.

### LIS1 as an antagonist of rapid, processive dynein motility: the ‘clutch’ model

The first in vitro study to assess the effect of LIS1 on dynein motility employed a microtubule gliding assay, in which dynein motors are nonspecifically adsorbed to the glass surface of an imaging chamber, followed by addition of free microtubules which are translocated across the surface by dynein ensembles. Addition of LIS1 resulted in a pronounced reduction in microtubule gliding velocity (Yamada et al., 2008), a finding that was confirmed in more recent studies (Baumbach et al., 2017; Torisawa et al., 2011; Wang et al., 2013). The first single-molecule study to assess the role of LIS1 utilized purified native mammalian brain dynein non-specifically adsorbed to plastic beads to track motor motility. Isolated dynein supports processive movement of these beads, albeit to a relatively low degree of processivity (0.7 µm - 2 µm; compare to 5–9 µm for activated DDA complexes) (Belyy et al., 2016; Mallik et al., 2004; McKenney et al., 2014; McKenney et al., 2010; Schlager et al., 2014; Vershinin et al., 2007). In this assay, LIS1 did not affect the instantaneous velocity of dynein-mediated bead translocation, although overall velocity was reduced due to increased bead pausing, suggesting the basis for reduced dynein-mediated microtubule gliding (in ensemble assays) is an increased fraction of paused motors engaged with the microtubule (McKenney et al., 2010). LIS1-induced pausing was also apparent when the dynein-bound beads were held under a resistive force in an optical trap. In these conditions, addition of LIS1 induced a high microtubule affinity state, in which the dynein-driven bead remained bound to the microtubule for prolonged periods of time under resistive load. Importantly, LIS1 itself did not interact with the microtubule, suggesting that the observed effects were a direct consequence of LIS1 binding to dynein. Additionally, LIS1 enhanced the ability of multiple dynein motors to move under load, suggesting that LIS1’s primary role may be to augment ensemble dynein motor function against hindering forces. This model could possibly account for a primary defect of lissencephaly, in which teams of dynein motors are compromised in their ability to translocate large cargos such as cell nuclei during INM or neuronal migration (see Figure 1). Subsequent studies have found that isolated mammalian dynein is in fact non-processive in fluorescent single-molecule assays (described above), and that attachment of the motor to surfaces such as glass coverslips or plastic beads presumably induces a conformational change that leads to minimal processive movement (Belyy et al., 2016), raising questions about the interpretation of these results. Given recent advances in the understanding of dynein’s mechanisms of autoinhibition and activation (discussed below), these data could conceivably be reinterpreted as a consequence of LIS1’s effect on the dynein autoinhibited conformation.

In stark contrast to human dynein, budding yeast dynein was observed to be a processive motor without the need for additional factors (Reck-Peterson et al., 2006), thus permitting the ability to interrogate the effects of Pac1 on dynein’s intrinsic motility parameters. These studies revealed that, similar to mammalian LIS1, Pac1 dramatically reduced dynein’s velocity in single-molecule assays by promoting a high microtubule binding affinity state of dynein (Huang et al., 2012; Toropova et al., 2014), consistent with the bead trapping assays described above. It was also noted in both mammalian and yeast systems that Pac1/LIS1 does not appear to dramatically impact dynein’s enzymatic activity (Huang et al., 2012; McKenney et al., 2010; Yamada et al., 2008); although, one study did observe a small but significant enhancement of ATP hydrolysis by dynein by LIS1 (Mesngon et al., 2006). These observations led to the model of Pac1 acting as a ‘clutch’, which, much like one in a car, disengages the engine (*i.e.*, ATPase activity) from the transmission (*i.e.*, for motility) (Huang et al., 2012). In contrast to the gliding assays described above, however, much higher levels of Pac1 were needed to achieve velocity reductions similar to those for the gliding assays. For instance, whereas equimolar to 3-fold molar excess of LIS1 was sufficient to induce bead pausing (McKenney et al., 2010) or reduce gliding velocity of mammalian dynein by ~75% (Yamada et al., 2008), respectively, a larger molar excess of Pac1 (12–300-fold) was required to achieve a similar velocity reduction (DeSantis et al., 2017; Huang et al., 2012). Recently published work has revealed that the ability of Pac1 to reduce dynein velocity may be due, at least in part, to spurious Pac1-microtubule binding in vitro, and not via direct Pac1-dynein binding (Marzo et al., 2020) (discussed below).

How might Pac1/LIS1 reduce dynein velocity and promote a microtubule bound state? Potential answers to these questions were provided in large part by cryoEM analysis of a dynein-Pac1 complex, which revealed two possibilities that are not necessarily mutually exclusive: (1) Pac1 binds within the AAA+ ring of dynein (near AAA3/AAA4), in a position that could partially block the docking of the linker element (which mediates the powerstroke) at its ‘resting’ site at AAA5 (Toropova et al., 2014). Preventing the linker from docking at AAA5 was proposed to prevent progression through the mechanochemical cycle, and to somehow lock the AAA+ ring in a conformational state that leads to high microtubule affinity. Consistent with this notion, it is well established that the nucleotide-bound state of dynein governs a series of conformational changes within the motor domain that in turn coordinate microtubule affinity (reviewed in Schmidt, 2015; Schmidt and Carter, 2016). (2) A subsequent study found that Pac1 may lock the AAA+ ring in a similarly high microtubule affinity state by clamping AAA3/4 and AAA5 together (DeSantis et al., 2017). It remains to be determined how these two models each contribute to affecting dynein-microtubule binding affinity, and more importantly, whether modulation of microtubule-binding affinity by Pac1 is indeed a relevant physiological activity.

In contrast to this mode of action, Pac1 was found to have the opposite effect on dynein motility when the AAA3 module – near Pac1-binding site 1 (see Figure 2C) – was mutated such that it could bind but not hydrolyze ATP (DeSantis et al., 2017; Kon et al., 2004). Such a dynein mutant moves at roughly 5% of wild-type speeds; however, addition of Pac1 led to a ~ 80% increase in the velocity of this mutant (to approximately 10% of wild-type dynein speed), and weakened its microtubule binding affinity (DeSantis et al., 2017). This mutation also led to the binding of a second Pac1 WD-40 domain near the stalk of the dynein motor domain, as apparent by cryoEM (Figure 2; site 2). It was thus posited that Pac1 binding may have contrary effects on dynein motility, depending on the nucleotide-bound state of the AAA3 module, which acts to convey the nucleotide-bound status of AAA1 (the main site for ATP binding and hydrolysis [Kon et al., 2004]) to conformational changes throughout the dynein motor domain (Bhabha et al., 2014; DeWitt et al., 2015; Nicholas et al., 2015). Although the authors noted that mutations in this newly discovered LIS1 binding-site led to a ~ 30% reduction in dynein localization to microtubule plus ends within yeast cells, the physiological relevance of this binding site for dynein function remains to be determined since functional in vivo studies have not yet been done.

How might promotion of a microtubule-bound state and/or reduction of dynein velocity support cellular dynein activity (*e.g.*, nuclear migration)? Several studies have determined that LIS1 is important for high-load dynein activities, in which dynein must overcome resistive forces to translocate cargoes (McKenney et al., 2010; Reddy et al., 2016; Yi et al., 2011). Stabilizing a microtubule-bound state could theoretically promote this behavior in cells. For example, injection of function-blocking LIS1 antibodies in rat cortical neurons inhibited axonal transport of large, but not small vesicular cargoes (Yi et al., 2011), although other studies have reported that depletion of LIS1 broadly impacts vesicular cargo transport in primary dorsal root ganglion neurons (Moughamian et al., 2013; Pandey and Smith, 2011). Another role for LIS1 in promoting high-load dynein transport comes from studies of dynein-mediated translocation of lipid droplets (LDs), which lend themselves to in vivo optical trapping due to their unique refractive properties. The authors noted that dynein motors bound to LDs dynamically responded to external load induced by the optical trap by increasing their force output, resulting in an enhanced probability of the LD ‘escaping’ the trap. However, depletion of LIS1, or its binding partner Nde1/Ndel1, eliminates this effect, indicating that these proteins plays a role in a dynein-mediated ‘force adaptation’ response (Reddy et al., 2016). Finally, LIS1 was also shown to improve the ability of DDA complexes to migrate through microtubule-bound tau condensates, suggesting that LIS1 enhances the ability of dynein-dynactin complexes to overcome obstacles (Tan et al., 2019). All of these results suggest that LIS1 could conceivably modulate the force-producing properties of dynein for efficient transport of diverse cargos in a crowded cellular environment.

As noted above, LIS1 and Pac1 have both been shown to be required for dynein’s microtubule plus end association in cells (Lee et al., 2003; Markus et al., 2009; Moon et al., 2014; Sheeman et al., 2003; Splinter et al., 2012). It has been postulated that Pac1 directly promotes yeast dynein plus end localization by stabilizing the high microtubule affinity state of dynein; in this model, Pac1/LIS1 locks dynein on to plus ends via the dynein microtubule binding domain (DeSantis et al., 2017; Huang et al., 2012; Roberts et al., 2014), from where dynein-dynactin complexes are offloaded to either cortical sites, or vesicular cargoes (Egan et al., 2012; Lenz et al., 2006; Markus and Lee, 2011). Investigations into the molecular mechanisms by which it does so in vitro have revealed a complicated picture for LIS1 in promoting this activity. Reconstitution studies with human proteins showed that LIS1 is not strictly required for dynein plus end binding; rather, LIS1 plays a role in enhancing dynein’s plus end association: *i.e.*, it shifts the balance from minus end-directed motility to plus end binding (Baumbach et al., 2017; Jha et al., 2017). Current in vitro and in vivo evidence indicate that dynein does not directly contact the microtubule plus end; rather, its plus end localization is mediated by dynactin (via the p150 subunit), which can associate with plus ends either directly, or through its interaction with the end-binding protein, EB1 (Duellberg et al., 2014; Jha et al., 2017; Lenz et al., 2006; Schuster et al., 2011; Splinter et al., 2012; Xiang et al., 2000; Yao et al., 2012; Zhang et al., 2003; Zhang et al., 2008). Thus, dynein likely does not directly contact the microtubule plus end lattice in cells, arguing against a role for LIS1 in locking dynein on to microtubule plus ends. In further support of this idea, the microtubule-binding domain of dynein is dispensable for plus end association in budding yeast cells (Lammers and Markus, 2015). It was also noted that overexpression of Pac1 does not lead to dynein binding along microtubules in cells, but rather increases the association of dynein with plus ends specifically (Markus et al., 2011). In addition to a direct plus end recruitment mechanism, studies indicate that kinesin (Kip2)-mediated transport of yeast dynein to the plus ends accounts for ~25% of dynein’s plus end-targeting (Carvalho et al., 2004; Markus et al., 2009). Consistent with these in vivo data, Pac1 links dynein to Bik1 (CLIP-170 homolog) and the plus end-directed kinesin in vitro (Roberts et al., 2014; Sheeman et al., 2003). Thus, at least in the context of plus end-targeting, LIS1 does not appear to function by modulating dynein-microtubule affinity. How then does LIS1 support dynein function in vivo? Recent evidence challenges the inhibitory model for LIS1 function, and instead suggests a role for LIS1 in activating dynein’s motility.

### LIS1 as activator of processive dynein motility: the ‘catalytic check valve’ model

Although it is difficult to rule out the possibility that one function of LIS1 is to promote a high microtubule affinity state of dynein, recent studies have posited a new model for the function of LIS1 and at least two of its orthologs: Pac1 (*S. cerevisiae*) and NudF (*A. nidulans*). Four new independent studies came to the same conclusion: that LIS1 promotes dynein activity by inducing and/or stabilizing its uninhibited, ‘open’ conformational state, which is likely a rate-limiting step in the assembly of the active dynein-dynactin-adapter (DDA) complex that is necessary for processive minus end-directed motility (Elshenawy et al., 2020; Htet et al., 2020; Marzo et al., 2020; Qiu et al., 2019; Zhang et al., 2017).

The first indication that LIS1 might enhance rather than inhibit dynein motility came from single-molecule experiments performed with assembled dynein-dynactin-cargo adapter (DDA) complexes. This is in stark contrast to those experiments described above, in which LIS1 (or Pac1) was assayed with isolated dynein. Although one of these studies observed that LIS1 had no effect on the velocity of DDA complexes (Jha et al., 2017), the other two found that addition of LIS1 led to a significant increase in their velocity (by as much as ~2.5 fold) (Baumbach et al., 2017; Gutierrez et al., 2017). Of note, these studies all observed varying degrees of co-migration of motile DDA complexes with LIS1 using multi-color single-molecule imaging. Mutational analysis indicated that LIS1 is indeed bound to dynein’s AAA+ motor domain during these movements (Gutierrez et al., 2017). Thus, direct binding of LIS1 to dynein motor domains within motile DDA complexes is not sufficient to stall or reduce the velocity of dynein as posited by the clutch model detailed above. These data raised the question as to how LIS1 binding to the AAA+ ring in a manner that potentially stabilizes a high microtubule affinity state could increase DDA velocity? One clue came from the surprising recent finding that the dynactin complex – which is built upon a small actin filament (Schafer et al., 1994) – has the potential to scaffold not only one (DDA complex), but two dynein dimers (D_2_DA complex) (Grotjahn et al., 2018; Urnavicius et al., 2018). As stated above, dynactin binding to dynein, which requires a cargo adaptor molecule such as BicD2, activates dynein motility by orienting the dynein motor heads in a parallel manner that is conducive for motility, and by enhancing the microtubule landing rate of the complex via the p150 subunit of dynactin (McKenney et al., 2014; McKenney et al., 2016; Nirschl et al., 2016). However, the finding that dynactin could also scaffold multiple dynein dimers within a single processive complex was quite surprising. Importantly, these D_2_DA complexes exhibit increased velocity and force output with respect to DDA complexes (Urnavicius et al., 2018). These findings, along with the observation that LIS1 stimulates DDA velocity, hinted at the possibility that LIS1 may stimulate the formation of D_2_DA complexes.

**Figure 3.**
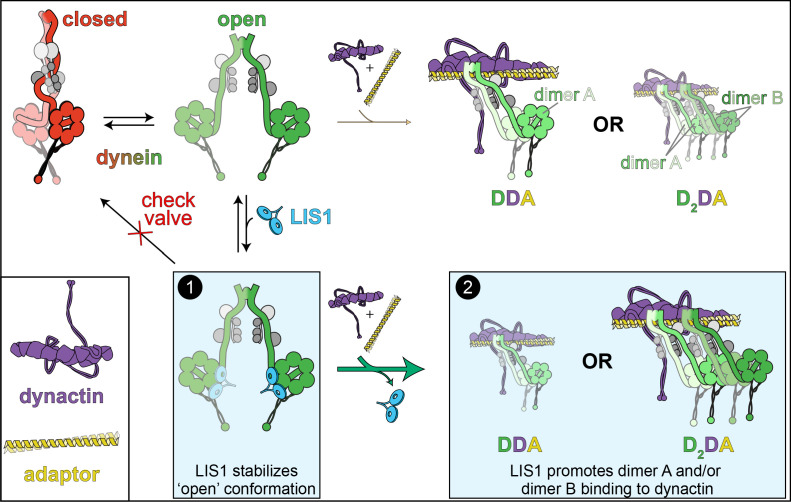
The ‘catalytic check valve’ model for LIS1 function. The new model for LIS1 function posits the following mode of action: dynein stochastically switches from closed (phi conformation, red), to open states (green; accessory chains shown in gray for both states) (Marzo et al., 2020). Adopting the latter state is likely a rate-limiting step in assembly of dynein-dynein-adaptor (DDA) complexes (Zhang et al., 2017). LIS1/Pac1 preferentially binds dynein in the open state (Htet et al., 2020; Marzo et al., 2020), and acts as a ‘check valve’ that prevents the return of dynein to the phi conformation (Box 1). In so doing, LIS1/Pac1 promotes assembly of active, processive DDA (with only one dimer, dimer A) and D_2_DA complexes (with dimers A and B). The probability of D_2_DA forming may be the product of the probability of a DDA complex assembling times itself (*p*DDA x *p*DDA); however, it is possible that the addition of the 2^nd^ dynein dimer (dimer B) occurs cooperatively due to the number of apparent contacts between the two dynactin-bound dynein dimers (Urnavicius et al., 2018), in which case the probability of D_2_DA assembly is greater than *p*DDA^2^. In either case, by increasing the probability of DDA complex assembly, addition of LIS1 also increases the probability of D_2_DA assembly, as has been observed in vitro (Elshenawy et al., 2020; Htet et al., 2020). In addition to stabilizing the open conformation, LIS1 also promote DDA/D_2_DA complex by an unknown mechanism that may involve LIS1-mediated dynein allostery, or linking dimer B to dimer A (Box 2; see text). It is interesting to note that LIS1 does not need to comigrate with dynein to affect DDA (or D_2_DA) motility; in fact, studies show that LIS1/Pac1 does not localize with dynein cargoes in cells, and may dissociate in a regulated manner prior to initiation of cargo transport (Jha et al., 2017; Lammers and Markus, 2015). In this way, LIS1 acts catalytically to increase DDA and D_2_DA assembly.

In two of the recent studies that employed mammalian proteins (Elshenawy et al., 2020; Htet et al., 2020), the authors found that addition of LIS1 increases the proportion of assembled DDA complexes, and increases the velocity and force output of these complexes by increasing the fraction of D_2_DA complexes (with respect to DDA complexes, Figure 3). Consistent with this latter notion, the LIS1-mediated velocity increase required that both of the dynein dimers have intact motor domains, as addition of truncated dynein tail complexes abrogated the LIS1-mediated velocity increase (Elshenawy et al., 2020). In addition to providing evidence for LIS1 promoting DDA/D_2_DA complex assembly through a mechanism that involves stabilization of the ‘open’ dynein conformation (see Figure 3; Box 1), these studies also found that LIS1 promotes complex assembly even when ‘open’ dynein mutants are used (see Figure 3, Box 2). Thus, LIS1 likely promotes dynein activity by at least two distinct mechanisms (see below).

Following with the conserved nature of this process, another of the recent studies surprisingly found that the phi conformation is conserved in budding yeast, and that the potential to adopt the autoinhibited state is inversely related to the run length of single dynein molecules (Marzo et al., 2020). This provided the authors with the ability to directly assess the role of Pac1 in promoting the ‘open’ state. Using single-molecule assays, they noted that addition of Pac1 increased the run length of wild-type dynein, but not ‘open’ dynein mutants, similar to some of the results reported for the mammalian homologues. In budding yeast, Pac1 is required for targeting dynein to microtubule plus ends and the cell cortex, and is consequently required for dynein activity (Lee et al., 2003; Markus et al., 2009; Sheeman et al., 2003). Consistent with a role for Pac1 in promoting release from, or preventing reversion back to the phi conformation, cortical targeting and activity of dynein in cells lacking Pac1 could be rescued with ‘open’ dynein mutants (Marzo et al., 2020). Finally, a study employing *A. nidulans* also revealed a critical role of NudF in cargo-adapter-mediated dynein activation in vivo, and that cells harboring the analogous 'open' dynein mutations bypass the NudF requirement for dynein-mediated cargo transport (Qiu et al., 2019). It is worth noting that in both fungal systems, open dynein mutants were not sufficient to completely rescue loss of cellular Pac1/NudF function (Marzo et al., 2020; Qiu et al., 2019), furthering the notion that LIS1 and its homologs promote dynein activity by additional, as yet unclear mechanisms. Taken together, all these data support a model whereby LIS1 aids in the activation of dynein by: (1) induction and/or stabilization of its ‘open’ state; and, (2) promoting DDA/D_2_DA complex assembly (Figure 3).

**Figure 4.**
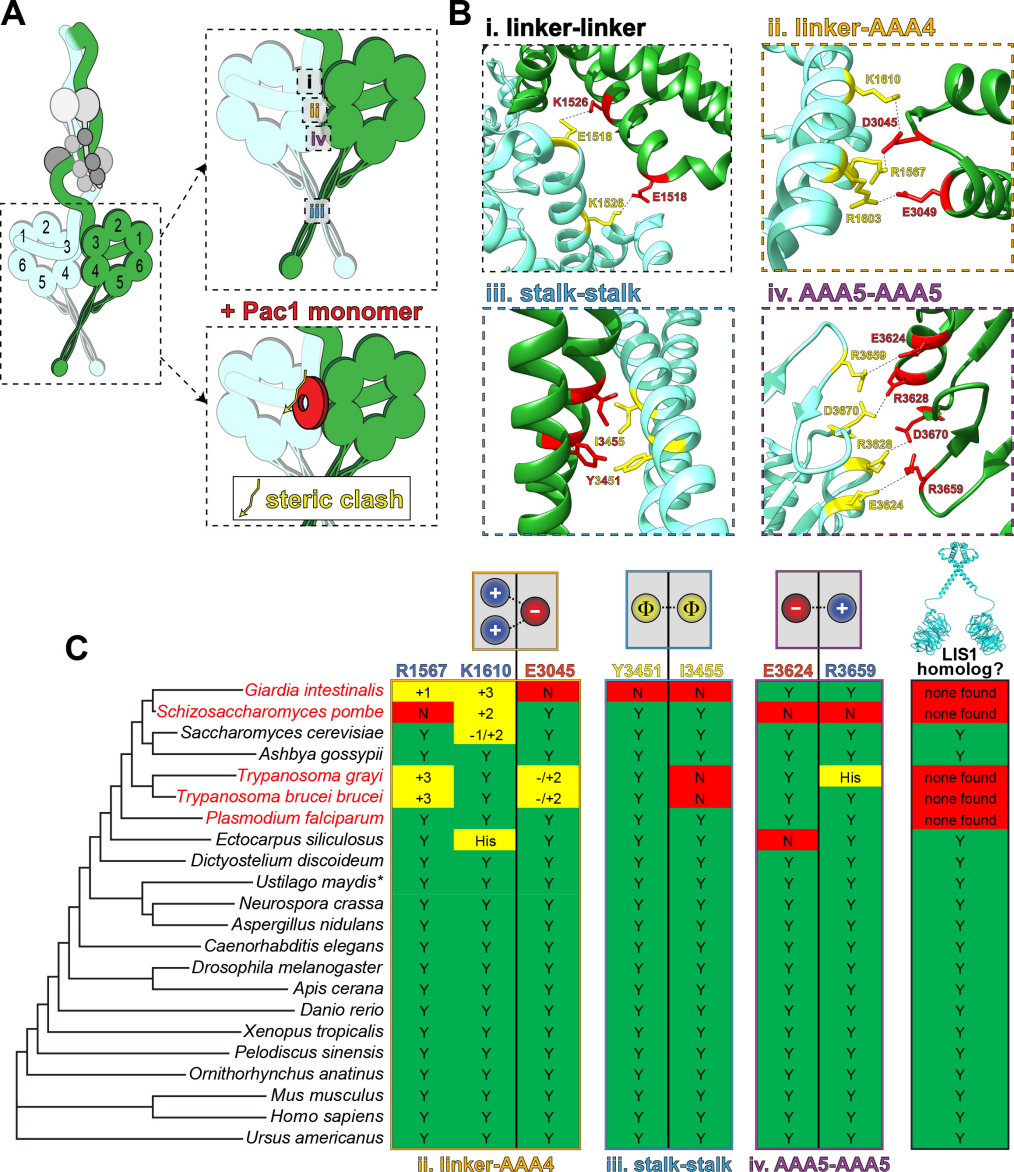
Dynein’s phi particle conformation and its potential coevolution with LIS1 function. (**A**) A cartoon model for the dynein phi particle (green and aquamarine, heavy chains with AAA domains numbered; gray, accessory chains), with the four intermolecular contact points identified by cryoEM indicated. Bottom panel illustrates the steric clash (indicated by jagged yellow line) between a dynein-bound LIS1/Pac1 monomer (red, bound to the dynein motor domain in green), and the 2^nd^ motor domain (in aquamarine; adapted from Marzo et al., 2020). (**B**) High resolution cryoEM structures of the four intermolecular contact points that stabilize the human dynein phi particle (adapted from Zhang et al., 2017). Note that regions ii, iii and iv have been experimentally validated, while region i has not yet been tested for its contribution to phi particle assembly (Marzo et al., 2020; Zhang et al., 2017). (**C**) Phylogenetic tree of cytoplasmic dynein heavy chains from the indicated species, and summary of the degree of their conservation (or lack thereof) for the three validated intermolecular contact points that stabilize the phi particle (only those sequences annotated as cytoplasmic dynein-1 homologs were selected for this analysis; see Figure 4—figure supplement 1 for sequence alignments of all four regions; *note that the dynein heavy chain from *Ustilago maydis* is encoded by two separate genes, *dyn1* and *dyn2*, which encode approximately 2/3^rd^ and 1/3^rd^ of the motor, respectively [Straube et al., 2001]). The residues listed above (for the human dynein heavy chain) are color coded according to their chemical properties, with their mode of interaction depicted in the cartoons (blue, positively charged residues, ‘+”; red, negatively charged residues, ‘-‘; yellow, hydrophobic residues, ‘Φ”). Degree of conservation for each residue is indicated as ‘Y’ (green, yes, conserved), ‘N’ (red, not conserved), or with a +/- value (yellow) indicating a potentially conserved residue up to three positions N- or C-terminally situated, respectively. ‘His’ indicates the presence of a histidine in these respective positions, which has the potential to form a salt bridge depending on the local microenvironment. The column on the right indicates whether a clear LIS1 ortholog was identified by sequence alignment or genome annotation (‘Y’, homolog present). Species names indicated in red are those in which no LIS1 homolog was identified. Note that the five species with no apparent LIS1 homolog exhibit a low predicted propensity to adopt the phi particle, as determined by the sequence alignments.

How might Pac1/LIS1 promote or stabilize dynein’s open conformation? Insights into this mechanism come from cryoEM structures of mammalian dynein in the autoinhibited phi state (Zhang et al., 2017), and a yeast dynein-bound Pac1 co-complex (DeSantis et al., 2017). Docking the latter model into the former revealed a large steric clash between Pac1 and the dynein motor domain to which Pac1 was not bound (Htet et al., 2020; Marzo et al., 2020), indicating that the phi conformation is incompatible with Pac1/LIS1 binding (Figure 4A). Consistent with this idea, both Pac1 and LIS1 were shown to bind dynein with higher affinity when the latter was in the ‘open’ state (Htet et al., 2020; Marzo et al., 2020). Taken together with the observation that single molecules of yeast dynein appear to stochastically adopt the autoinhibited state (Marzo et al., 2020), the data suggest a model whereby LIS1 could be an opportunistic binder: upon dynein stochastically switching into its ‘open’ state, LIS1 binding to dynein prevents its reversal back into the phi conformation. In this new model, LIS1 acts as a ‘catalytic check valve’, acting as both a catalyst for the assembly of DDA and D_2_DA complexes, and a check valve that prevents the reverse ‘flow’ of dynein back into the autoinhibited phi conformation. Once it is ‘open’, assembly of dynein into the DDA (or D_2_DA) complex occurs more readily (Zhang et al., 2017). This model is consistent with earlier observations that LIS1 could enhance the formation of dynein-dynactin complexes in *Drosophila *and *Xenopus* egg lysates (Dix et al., 2013; Wang et al., 2013). In support of a catalytic mode of action, it was noted that LIS1 does not need to remain bound to the D_2_DA complexes to sustain higher velocities (Elshenawy et al., 2020; Htet et al., 2020). Dissociation of LIS1 from the fully assembled D_2_DA complex therefore ‘regenerates’ a LIS1 molecule such that it could conceivably act again in another round of D_2_DA assembly. These data are consistent with in vivo data from budding yeast and filamentous fungi that suggest that LIS1 dissociates from dynein-dynactin complexes prior to, coincident with, or shortly after initiation of transport (Egan et al., 2012; Lee et al., 2003; Lenz et al., 2006; Markus and Lee, 2011). In fact, dissociation of DDA/D_2_DA complexes from LIS1 might be an important step in the initiation of dynein-mediated transport. Studies in budding yeast suggest that adaptor binding might play a role in triggering dissociation of LIS1 from dynein, and moreover, that preventing their dissociation leads to defects in dynein-mediated nuclear migration in this model system (Lammers and Markus, 2015; Markus et al., 2011). A similar adaptor-mediated dissociation of dynein from LIS1 has also been observed in vitro with human proteins (Jha et al., 2017), and may account for the varying degrees of DDA-LIS1 colocalization in single-molecule assays (Baumbach et al., 2017; Elshenawy et al., 2020; Gutierrez et al., 2017; Htet et al., 2020; Jha et al., 2017).

**Figure 4—figure supplement 1.**
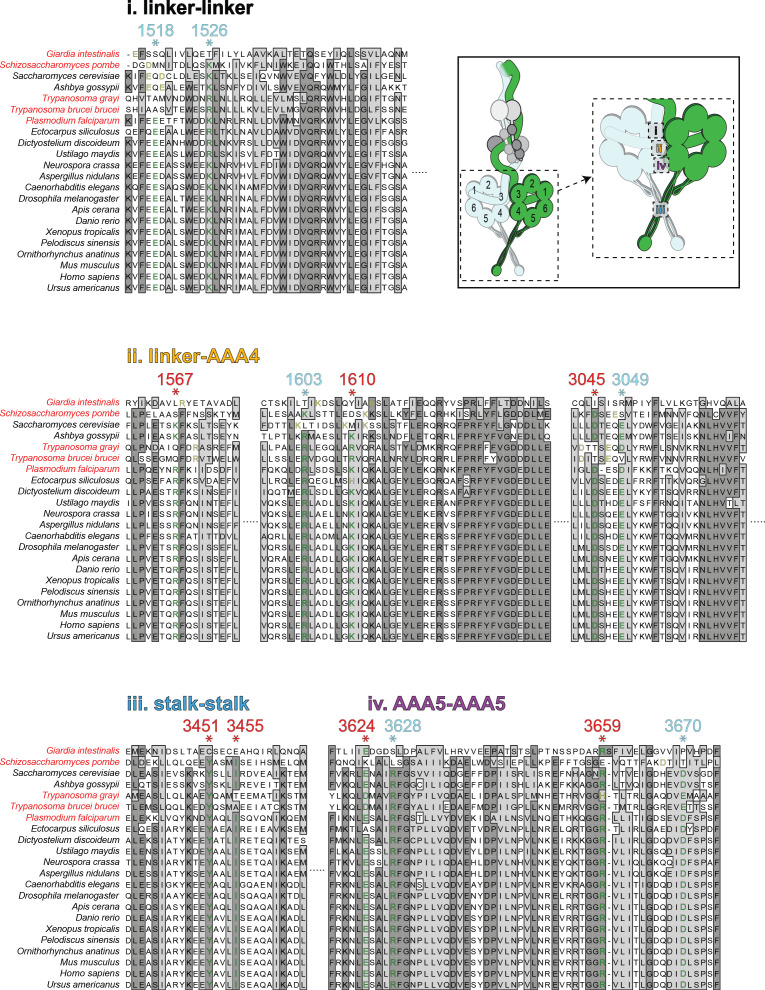
Sequence alignments of the intermolecular contact regions for cytoplasmic dyneins from disparate species. Alignments of the four predicted intermolecular contact points that stabilize the phi particle conformation (listed in order from surfaces 1 through 4, as indicated). Cytoplasmic dynein-1 heavy chain sequences were identified based on sequence annotations. The numbered residues above each line (with *) denote the residues that mediate the intermolecular interactions (red and cyan depict residues experimentally validated, and those not yet tested, respectively; those in red were used to generate the table shown in Figure 4C). See Figure 4B for precise predictions of interacting residues. For each starred residue, a conserved residue in each ortholog is denoted in green, and potentially conserved residues (offset by up to three positions in the N- or C-terminal direction) are denoted in yellow. If no potentially conserved residue was identified, the residue is denoted in black. Species names in red indicate those species with no apparent LIS1 homolog.

As noted above, LIS1 also promotes D_2_DA complex assembly in a manner that does not rely on stabilizing the ‘open’ conformation (see Figure 3; Box 2; Elshenawy et al., 2020; Htet et al., 2020). It is conceivable that LIS1 directly promotes the binding of dynein to dynactin (*i.e.*, through an allosteric mechanism), or that LIS1 affects the binding of only the second dynein dimer (see Figure 3; ‘dimer B’) to dynactin. Although additional studies will be required to determine how LIS1 might do so, one potential mechanism might involve the dynein dimer A-bound LIS1 recruiting dynein dimer B to dynactin (by acting as a bridge between these two dynein dimers). To be clear, this mechanism is distinct from LIS1’s role in stabilizing the open state, since a monomeric LIS1 fragment – which only possesses one dynein-binding site – is sufficient to promote DDA/D_2_DA complex assembly (Htet et al., 2020) and can partially rescue loss of Pac1 when overexpressed in yeast (Huang et al., 2012). Given a LIS1 monomer is likely sufficient to stabilize the open state (see Figure 4A), this fragment would be expected to stimulate DDA and D_2_DA complex assembly. However, whether the monomeric LIS1 fragment can specifically promote D_2_DA assembly when ‘open’ mutants are used (thus bypassing LIS1’s role in stabilizing the ‘open’ state) – in spite of its ability to only bind one dynein dimer – remains an open question. Another possibility, as posited by Htet et al., could be that by slowing down its velocity, the likelihood of dimer B being added to a motile DDA complex is increased. Given the variable reports for a dynein-bound LIS1 in affecting DDA velocity (from increase [Baumbach et al., 2017] to no change [Gutierrez et al., 2017] to a small reduction [Elshenawy et al., 2020; Htet et al., 2020]) and the small velocity reduction noted for these two latter studies (<20%), this mode of action for LIS1 seems unlikely.

How can we reconcile the apparent Pac1/LIS1 inhibitory activity described above with these new data? Marzo et al. noted that Pac1 robustly binds microtubules in the experimental conditions that were used for prior Pac1-dynein studies (DeSantis et al., 2017; Huang et al., 2012; Toropova et al., 2014) – an observation that had not been previously noted. Given that Pac1 does not appear to bind microtubules in cells, even upon its overexpression (Markus et al., 2011), the authors of this study sought to determine whether the Pac1-microtubule binding was the reason for the velocity reduction phenotype. The first indication that this might be the case was that the Pac1 velocity reduction effect did not require that Pac1 be stably bound to dynein. Specifically, Marzo et al. noted that those dynein complexes that comigrated with Pac1 moved with the same reduction in velocity as those that were not bound to Pac1 (note that all dyneins still encountered microtubule-bound Pac1 molecules). Then, by using two different means to reduce Pac1-microtubule binding – increasing ionic strength, and inclusion of cell extracts – the authors noted that the degree of microtubule binding by Pac1 directly correlates with its ability to reduce dynein velocity. Although a small Pac1-mediated velocity reduction (~20%) was still observed in the highest ionic strength buffer tested, it remains unclear whether this is due to residual Pac1-microtubule binding (which was observed in these conditions), or a bona fide Pac1 inhibition of dynein’s linker swing as posited by the clutch model (Toropova et al., 2014). It is worth noting that the related studies using mammalian proteins also observed a similar small velocity reduction of motile DDA complexes that were bound to LIS1 (Elshenawy et al., 2020; Htet et al., 2020), which contrasts with previous findings (Baumbach et al., 2017; Gutierrez et al., 2017). One of these studies noted a small degree of LIS1-microtubule binding, which could potentially account for the velocity reduction (Elshenawy et al., 2020). However, given the degree of this effect (<20% reduction in velocity), it is difficult to envision how this activity may directly promote DDA/D_2_DA complex assembly, microtubule plus end-binding, or other localization phenotypes. Future studies will be needed to determine whether the small velocity reduction effect is indeed physiologically relevant, and how it may support dynein-mediated transport functions.

## Coevolution of LIS1 with dynein autoinhibition

A prediction of this new model for LIS1 function is that its activity would be required in organisms in which dynein adopts the phi conformation. To determine whether this might be the case, we used a phylogenetic approach to predict whether dynein motors from various species adopt the autoinhibited state, and then correlated this predicted propensity with the presence or absence of a LIS1 homolog. We focused on those residues that were experimentally determined to stabilize the dynein phi conformation (see Figure 4A and B, and Figure 4—figure supplement 1; at surfaces ii through iv; numbering for human dynein): R1567, K1610, E3045, Y3451, I3455, E3624, and R3659 (Marzo et al., 2020; Zhang et al., 2017). Potential LIS1 homologs were identified by sequence alignment, and by identification of two invariant residues among LIS1 homologs that have been shown to be important for dynein binding (Gutierrez et al., 2017; Toropova et al., 2014): R316 and W340 (see Figure 2D). This revealed that cytoplasmic dynein-1 heavy chain sequences from most species are predicted to have the capacity to adopt the phi conformation, with only four notable exceptions: *Giardia intestinalis*, *Schizosaccharomyces pombe*, *Trypanosoma grayi*, and *Trypansoma brucei brucei* (Figure 4C). Interestingly, all but one of the species with conserved dynein intermolecular contacts possess a clear LIS1 homolog (the unicellular protist *Plasmodium falciparum*). On the other hand, all four of those species that contain dynein heavy chains lacking the phi particle contact residues have no apparent LIS1 homolog. These data suggest that the autoinhibited conformation of dynein may have coevolved with LIS1, and further indicate that the reliance on LIS1 for dynein function strongly correlates with the presence of the autoinhibited state.

Cytoplasmic dynein-2, the retrograde motor that transports cargo within flagella and cilia (intraflagellar transport, or IFT), also adopts the phi conformation in vitro (Toropova et al., 2017; Toropova et al., 2019) and in vivo (Jordan et al., 2018). There is currently little evidence that LIS1 plays a role within cilia, although localization of mammalian LIS1 to motile cilia has indeed been reported (Pedersen et al., 2007). The authors of this study also identified a LIS1-related protein that is present in the *Chlamydomonas* flagellum (CrLis1; 30% identity/45% similarity with human LIS1), although it should be noted that CrLis1 lacks the two invariant residues discussed above (R316 and W340). Therefore, it is possible that LIS1 or LIS1-like proteins play similar roles for dynein motors specialized for cilia function. Further research into the autoinhibition and activation mechanisms of ciliary dyneins will be of high interest in the future.

## Reconciling new model with old findings

In light of this new model for LIS1 function, the precise molecular mechanism by which it affects the various cellular and developmental processes in which it has been implicated needs to be reevaluated. For example, this newly described function for Pac1 addresses long unanswered questions in the yeast dynein field. It has been previously observed that dynein-dynactin complex assembly takes place at microtubule plus ends (Markus et al., 2011; Moore et al., 2008), a site where Pac1-bound dynein molecules localize (Lee et al., 2003). However, why the plus end was a platform for dynein-dynactin complex assembly has long been a mystery. The catalytic check valve model now answers this question, and indicates that Pac1-bound dynein molecules – which associate with plus ends via Bik1/CLIP-170 (Sheeman et al., 2003) – are in the ‘open’ state, and thus primed for interaction with dynactin. Offloading of these preassembled complexes to cortical Num1 receptors, which requires intact dynein-dynactin complexes (Lee et al., 2003; Markus and Lee, 2011; Tang et al., 2012), is therefore perfectly suited to follow plus end-association of Pac1-dynein complexes.

An analogous situation is likely at play for LIS1-dependent localization of dynein to its numerous cellular sites in higher eukaryotic cells, most of which have been shown to require dynactin (*e.g.*, microtubule plus ends, nuclear envelope, kinetochores [Coquelle et al., 2002; Faulkner et al., 2000; Moon et al., 2014; Raaijmakers et al., 2013; Splinter et al., 2012]). In the case of targeting dynein to plus ends, the role of LIS1 could be easily explained by this new model. In human model systems, the association of dynein with microtubule plus ends is mediated by dynactin, either directly, or indirectly through dynactin’s association with EB1 (Duellberg et al., 2014; Jha et al., 2017). Thus, any condition in which dynein-dynactin interaction is enhanced (*e.g.*, by addition of LIS1) will naturally promote dynein plus end-association, as has been observed in vitro (Baumbach et al., 2017; Jha et al., 2017). Similarly, recruitment of dynein to its various other cellular sites relies on adaptor proteins (*e.g.*, BicD2), which interact only with dynein-dynactin, and not either complex alone (Splinter et al., 2012). Thus, depletion of LIS1 – or loss-of-function mutations – would be expected to reduce dynein-dynactin complex assembly, and consequently compromise localization and cargo transport. Heterozygous mutations of LIS1 are causative of lissencephaly (*i.e.*, LIS1 exhibits haploinsufficiency), suggesting that cellular concentrations of LIS1, and thus activated dynein, are critically tuned for specific cellular processes.

Given the role of LIS1 in promoting assembly of D_2_DA complexes (see Figure 3), loss-of-function LIS1 mutants would also be expected to compromise dynein force output (Elshenawy et al., 2020; Urnavicius et al., 2018), as has been observed in cultured cells microinjected with LIS1 function-blocking antibodies (Yi et al., 2011), or depleted of LIS1 (Reddy et al., 2016). Reduced D_2_DA assembly would also compromise dynein’s ability to navigate the crowded microenvironment of MAP-decorated microtubules. One such MAP, tau, forms condensates on microtubules that can induce pausing of DDA complexes. LIS1 augments the ability of dynein to bypass tau condensates, possibly as a consequence of promoting assembly of D_2_DA complexes (Tan et al., 2019).

Another important prediction of the catalytic check valve model is that LIS1 would be expected to increase the microtubule binding affinity for dynein alone, since the ‘open’ mutants exhibit a higher microtubule association rate than wild-type dynein (Zhang et al., 2017). This in turn could conceivably increase the rate of ATP hydrolysis given the role of microtubule-binding in the progression of the mechanochemical cycle. Lending further support to the catalytic check valve model, LIS1 has indeed been shown to increase the microtubule landing rate of purified dynein (*i.e.*, it’s association rate) (Baumbach et al., 2017; Htet et al., 2020); however, its effects on dynein’s microtubule-stimulated ATPase rate have been variable, with most studies reporting little to no effect (McKenney et al., 2010; Yamada et al., 2008), and one reporting a significant increase (Mesngon et al., 2006). Given the data support a stochastic switching between ‘closed’ and ‘open’ states (Marzo et al., 2020), it is tempting to speculate that the increased microtubule affinity of dynein imparted by LIS1 in the optical trap (McKenney et al., 2010) is also a consequence of LIS1 stabilizing the ‘open’ state; moreover, this same reasoning could potentially account for the observed reduction in microtubule gliding velocity (Baumbach et al., 2017; Wang et al., 2013; Yamada et al., 2008). Since the phi conformation – which has been shown to be labile over time and experimental conditions in vitro – locks dynein’s linker domain in a pre-powerstroke state (Zhang et al., 2017), it will be important in the future to carefully determine how dynein’s enzymatic cycle is affected by the autoinhibition/activation cycle.

This new model also accounts for the nuclear and neuronal migration defect that is causative of lissencephaly. Either by disrupting dynein localization (*e.g.*, to cortical sites) or by reducing the number of motile DDA or D_2_DA complexes, loss-of-function LIS1 mutations or reduced cellular levels of LIS1 (due to heterozygous mutations) would globally impact dynein activity. Thus, loss-of-function LIS1 mutations would be expected to phenocopy loss-of-function dynein mutations. Surprisingly, however, dynein mutations correlate better with polymicrogyria, and not lissencephaly (Laquerriere et al., 2017; Poirier et al., 2013; Vissers et al., 2010; Willemsen et al., 2012). Although polymicrogyria (excessive number of abnormally small gyri) is also a neuronal migration disorder, the morphological differences between the diseased brains suggest a somewhat different underlying disease etiology. The reasons for this are unclear, but may be due to a role for LIS1 in other cellular processes that occur during early brain development. For instance, in addition to affecting dynein function, LIS1 has also been implicated in regulating actin-based processes (Chhatre et al., 2020; Kholmanskikh et al., 2003).

## Future challenges

Given this torrent of new data that have led to the revised model for LIS1 function in the dynein pathway, what are the next questions pertaining to dynein regulation to be answered? One immediate challenge is to more fully understand how LIS1 facilitates the formation of DDA and D_2_DA complexes, which will no doubt be aided by electron microscopy-based imaging methods. For instance, a high-resolution image of LIS1 bound to an open dynein complex will provide additional evidence for the check valve model. As stated above, additional experiments will also be needed to understand how LIS1 promotes D_2_DA assembly beyond simply stabilizing the ‘open’ conformation. Moreover, whether additional factors play a role in supporting LIS1 function is unclear. Since WD-40 domains often act as multi-protein assembly scaffolds, perhaps LIS1 function can be facilitated by other available surfaces of the beta-propeller once bound to the dynein motor domain. It is possible that other regions of the dynein-bound LIS1 beta-propeller recruit additional factors to further promote (or hinder) DDA/D_2_DA assembly. The role of LIS1 in other molecular pathways also remains to be more clearly established (*e.g.*, phagocytosis, actin polymerization [Chhatre et al., 2020; Kholmanskikh et al., 2003]). For instance, is LIS1’s role in the PAF-AH pathway truly orthogonal to its functions with dynein, or does PAF signaling play a role in dynein function, as has been suggested by at least one study (Ding et al., 2009)?

One of the most pressing questions to address is the role of the LIS1 and dynein-binding proteins Nde1/Ndel1 (also known as NudE/NudEL). The fungal homologues of mammalian Nde1/Ndel1 were first identified in *N. crassa* (RO-11) (Minke et al., 1999) and *A. nidulans* (NudE) (Efimov and Morris, 2000), and were shown to directly bind to LIS1 and participate in the nuclear migration pathway in these organisms. Of interest, the *nud*E gene in *Aspergillus* was isolated as a multicopy suppressor of a temperature-sensitive *nud*F (LIS1 homolog) mutant (Efimov and Morris, 2000). Mammals evolved two paralogues of these proteins, Nde1 (formerly NudE) and Ndel1 (formerly NudEL), which have also been shown to bind directly to LIS1 and dynein, and to participate in mammalian brain development (Feng et al., 2000; Niethammer et al., 2000; Sasaki et al., 2000). These proteins contain a conserved elongated N-terminal coiled-coil domain that directly interacts with LIS1 and the dynein intermediate chain (DIC) (Sasaki et al., 2000; Wang and Zheng, 2011; Zyłkiewicz et al., 2011). An unstructured and alternatively spliced C-terminal domain has also been reported to interact with the dynein heavy chain and light chain (McKenney et al., 2011; Nyarko et al., 2012; Sasaki et al., 2000). Thus, the interactions required for assembly of the tripartite dynein-LIS1-Nde1/Ndel1 complex are poorly understood, and additional information is needed to more fully understand its organization, and the nature and contribution of each of these interactions.

How does Nde1/Ndel1 affect dynein-LIS1 function? There is strong biochemical evidence that Nde1/Ndel1 interacts with both LIS1 and dynein simultaneously (Huang et al., 2012; McKenney et al., 2010; Wang et al., 2013; Zyłkiewicz et al., 2011) and plays a role in coordinating dynein-LIS1 binding (McKenney et al., 2010). These interactions are thought to occur through the binding of the Nde1/Ndel1 N-termini with DIC and LIS1 (and possibly the Nde1/Ndel1 C-termini with the dynein light chain, LC8 [McKenney et al., 2011]). It has been hypothesized that the LIS1 WD-40 domain directly contacts dynein’s motor domain while being simultaneously bound to Nde1/Ndel1 (McKenney et al., 2010; Tarricone et al., 2004), and thus Nde1/Ndel1 are well positioned to link LIS1 to dynein. While it is clear that a tripartite complex of Nde1/Ndel1, dynein, and LIS1 can be biochemically isolated, it should be noted that the simultaneous engagement of all three of these contact surfaces (*i.e.*, dynein motor-LIS1, DIC-Nde1/Ndel1, and Nde1/Ndel1-LIS1) has yet to be formally demonstrated. In fact, it is conceivable that such a complex could be formed without simultaneously engaging all three contact surfaces. These interactions, and the ability of Nde1/Ndel1 to recruit LIS1 to dynein in single-molecule assays, has led to the model that Nde1/Ndel1 act as a scaffold for the recruitment of LIS1 to dynein. Evidence from *A. nidulans* indeed suggest a role for NudE in promoting cargo adaptor-mediated dynein activation, at least in part by stabilizing and/or inducing the open conformation of dynein (Qiu et al., 2019), which would be an expected outcome of enhanced dynein-LIS1 binding. However, the nature of the tripartite dynein-LIS1-Nde1/Ndel1 complex and its assembly, and how Nde1/Ndel1 affects LIS1’s ability to catalyze DDA/D_2_DA assembly needs to be addressed using the expanded single-molecule, biochemical, and structural biology toolkits that have been recently developed.

Adding an additional layer of complexity, the unstructured N-terminus of the DIC serves as a binding platform for both Nde1/Ndel1 and the p150^Glued^ subunit of dynactin, which compete with each other for this binding site on the dynein complex (McKenney et al., 2011; Nyarko et al., 2012; Siglin et al., 2013). The role of the DIC-p150^Glued^ interaction in the assembly of DDA/D_2_DA complexes remains unclear due to this surface’s conspicuous absence from recent cryoEM structures of the dynein-dynactin structure (Urnavicius et al., 2018; Urnavicius et al., 2015). However, the competitive binding of Nde1/Ndel1 and p150^Glued^ for DIC suggests a potentially additional role for Nde1/Ndel1 in regulating the dynein-dynactin interaction beyond coordinating dynein-LIS1 binding. Further work will be needed to understand the sequence of interactions that occur for these numerous factors that ultimately leads to DDA/D_2_DA assembly, any structural changes that occur as a consequence of the multitude of interaction surfaces coming together, and finally, how these events ultimately result in proper dynein regulation within cells.

It is interesting to note that mutations in human NDE1 are causative of microcephaly, not lissencephaly (Alkuraya et al., 2011; Doobin et al., 2016), revealing that these proteins likely have distinct roles during brain development. Additionally, evidence indicates that Nde1/Ndel1 can interact with a large number of binding partners, suggesting their roles are not restricted to dynein regulation (Houlihan and Feng, 2014). Nonetheless, the affinity of LIS1 for Nde1/Ndel1 is high (in the nanomolar range [Tarricone et al., 2004]), suggesting that the Nde1/Ndel1-LIS1 complex is highly relevant in cell physiology. The mysterious roles of these proteins, and potentially other LIS1-interacting proteins, and how they impinge on LIS1’s ability to regulate dynein activity represent a tantalizing next step in our understanding of this complicated motor’s regulation in cells and human disease.
